# Rhizosphere Microorganisms Supply Availability of Soil Nutrients and Induce Plant Defense

**DOI:** 10.3390/microorganisms12030558

**Published:** 2024-03-11

**Authors:** Wannaporn Thepbandit, Dusit Athinuwat

**Affiliations:** 1 Faculty of Science and Technology, Thammasat University, Pathum Thani 12121, Thailand; w.thepbandit@gmail.com; 2 Center of Excellence in Agriculture Innovation Centre through Supply Chain and Value Chain, Thammasat University, Pathum Thani 12121, Thailand

**Keywords:** biological control agents, plant immune, plant elicitor, integrated plant disease, infection

## Abstract

Plant health is necessary for food security, which is a key determinant of secure and sustainable food production systems. Deficiency of soil nutrients and invasion of plant pathogens or insects are the main destroyers of the world’s food production. Synthetic fertilizers and chemical-based pesticides are frequently employed to combat the problems. However, these have negative impacts on microbial ecosystems and ecosystem functioning. Rhizosphere microorganisms have demonstrated their potency to improve or manage plant nutrients to encourage plant growth, resulting in increased yield and quality by converting organic and inorganic substances around the rhizosphere zone into available plant nutrients. Besides regulating nutrient availability and plant growth enhancement, rhizobacteria or fungi can restrict plant pathogens that cause disease by secreting inhibitory chemicals and boosting plant immunity to combat pests or pathogens. Thus, rhizosphere microorganisms are viewed as viable, alluring economic approaches for sustainable agriculture as biofertilizers and biopesticides. This review provides an overview of the role of rhizosphere microorganisms in soil nutrients and inducing of plant defenses. Moreover, a discussion is presented surrounding the recent consequences of employing these microorganisms and a sustainable strategy towards improving fertilization effectiveness, and encouraging stronger, more pest-resistant plants.

## 1. Introduction

The world population is estimated to extend to around 10 billion in 2050 and global food demand will increase by 60% [1]. This suggestion will contribute to significant challenges regarding adequate farm-based food production to feed and supply a growing population, and the limited availability of additional agricultural land resulting from urbanization [2]. Improvements in crop yields and reducing negative impacts, abiotic stress, plant pest, and diseases of food production, at the same time have been essential to solve the problems. In many situations, enhanced agricultural productivity or pest-disease control depend on the application of synthetic fertilizers and pesticides that are often unaffordable to many farmers and can have negative effects on humans, wildlife, the environment, ecological diversity, and others. Thus, it is imperative to investigate and implement sustainable methods that enhance agricultural food production in a manner that is more harmonious with the environment. Advancements in sustainable crop improvement, coupled with the adoption of eco-friendly agricultural practices, represent a transformative approach for modern farming systems which integrates several environmentally friendly techniques using natural resources [3]. These innovations not only aim to increase yield and efficiency but also emphasize the reduction of environmental impact, ensuring a balance between agricultural productivity and ecological preservation.

Plants are closely related to microorganisms in rhizosphere soil, such as those that provide nutrients–fertilizer, modify the physical and chemical properties of soil, and the manipulation of hormone signaling [4,5]. These are essential in determining plant health and can prevent plant diseases from biotic or abiotic stresses through rhizodeposition [6]. The rhizosphere zone represents a slot of microbial interaction that is established upon the recognition of the plant root system. This region generates carbon fixed through photosynthesis and an array of compounds including microbial secondary metabolites, carbohydrates, amino acids, and vitamins, all of which play a role in enhancing soil fertility [7,8]. It is a critical source of energy input in plant–soil ecosystems that improves the diversity of plant–microbe interaction. The primary result of which is the development of unique plant root microbial communities. Thus, understanding microbial diversity and their functions are a part of sustainable agriculture practice.

Rhizosphere microorganisms refer to the diverse array of microscopic lifeforms that inhabit the rhizosphere, the narrow region of soil that is directly influenced by root secretions and associated soil microorganisms. This region is characterized by a high level of biological activity due to the presence of substances secreted by roots, such as sugars, amino acids, organic acids, and various secondary metabolites. These substances serve as nutrients for microorganisms, fostering a rich and dynamic microbial community around plant roots. The composition of rhizosphere microorganisms is varied and includes several key groups including bacteria, fungi, protozoa, archaea, viruses (phages), and nematodes [9,10]. These microorganisms engage in a wide range of interactions with plant roots and each other, including symbiosis, competition, predation, and parasitism. Beneficial interactions, such as those between mycorrhizal fungi or nitrogen-fixing bacteria and plant roots, enhance plant nutrient acquisition and stress tolerance. In the rhizosphere, bacteria stand out as one of the most abundant and varied groups of microorganisms. They are pivotal in nutrient cycling processes such as nitrogen fixation and phosphorus and potassium solubilizations, in addition to playing key roles in suppressing diseases and fostering plant growth through the synthesis of various hormones and enzymes [11,12]. Fungi are integral to this ecosystem, with symbiotic mycorrhizal fungi forming advantageous connections with plant roots to enhance water and nutrient absorption, while free-living fungi aid in the decomposition of organic matter and further nutrient cycling [13]. Protozoa, the single-celled eukaryotes that prey on bacteria and other microorganisms, are crucial for the regulation of microbial populations and the cycling of nutrients in the rhizosphere [14]. Archaea, although similar to bacteria, are a genetically distinct group of microorganisms involved in critical processes such as ammonia oxidation, a vital component of the nitrogen cycle [15]. Viruses, particularly phages that infect bacteria, are present in the rhizosphere as well, where they impact the dynamics of microbial communities through predation and the transfer of genes [16]. Additionally, nematodes and other microfauna, though not strictly microorganisms, play significant roles in the rhizosphere by contributing to nutrient cycling and the dynamics of the soil food web, highlighting the complexity and interconnectedness of life in this unique soil environment [17].

The unique soil environment facilitates a myriad of ecological interactions among microorganisms, influencing community taxonomy [18]. The taxonomy of microbial communities involves categorizing and understanding the diverse array of microorganisms present in a given environment based on their evolutionary relationships and characteristics [19]. This classification extends from broad groups to more specific categories, following a hierarchical structure typically including domain, kingdom, phylum, class, order, family, genus, and species. In the scope of microbial communities, taxonomy provides a framework for organizing this diversity and understanding the roles and interactions of different microorganisms within the community. For example, bacterial phyla such as Bacteroidetes and Proteobacteria are enriched in the rhizosphere, whereas phyla like Chloroflexi and Acidobacteria are more abundant in bulk soil. This variation is influenced by factors such as plant type, soil conditions, and agricultural practices [20,21]. A recent study showed that protists in the rhizosphere might protect plants by feeding on other microorganisms and shifting the taxonomic and functional composition of microorganisms of the rhizosphere [22]. Likewise, the study of Zhang et al. [23] showed that rhizospheric protists have the potential to influence bacterial and fungal communities to influence the rhizospheric co-occurrence relationships of soybean plants. Furthermore, identifying a microorganism’s taxonomic position can provide clues about its physiological properties [24].

The physiological features of microorganisms encompass a wide range of functional attributes that enable their growth, survival, and behavior in diverse environments. These features include a variety of metabolic pathways, allowing them to utilize different sources of carbon and energy through processes such as aerobic and anaerobic respiration, fermentation, photosynthesis, chemolithotrophy, and methanogenesis [25,26]. Microorganisms also have specific mechanisms for nutrient uptake and assimilation, crucial for processing carbon, nitrogen, phosphorus, sulfur, and various trace elements and vitamins [27]. Their growth is influenced by optimal conditions related to temperature, pH, and salinity, with adaptations that allow some to thrive in extreme conditions, resisting desiccation, high salinity, and extreme temperatures [28]. Communication is another key physiological feature, with many microorganisms using chemical signals for quorum sensing to regulate collective behaviors like biofilm formation [29]. Mobility is facilitated by structures like flagella, cilia, or pili, enabling movement in response to environmental cues [30]. Whereas fungi have developed unique strategies for spreading and colonizing new environments by spore dispersal and hyphy growth [31]. Energy storage mechanisms allow for the accumulation of resources in forms such as glycogen, polyphosphate, or sulfur globules. To defend against threats, microorganisms can produce antibiotics, toxins, and form biofilms, and have developed various resistance mechanisms. Lastly, the cellular structure, including cell wall composition and the presence of specific organelles or appendages, plays a crucial role in their physiological capabilities.

**Figure 1:**
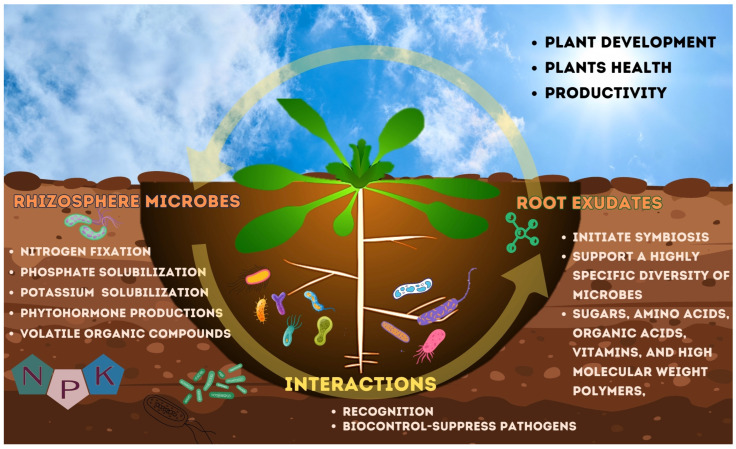
Schematic overview of benefits effect of plant interaction with microorganisms in the rhizosphere zone.

Beneficial rhizosphere fungal and bacterial communities are well-known as plant growth promoting microorganisms (PGPM). These microorganisms can colonize plant roots and support their hosts by producing phytohormones, improving soil nutrients, combatting pathogens infection, and tolerance to biotic and abiotic stress, leading to a reduction in the use of pesticides and synthetic fertilizers in crop [32,33]. Host plants provide rhizo-deposit nutrients, boundary cells, mucilage, lignin, and cellulose as sources of food for microorganisms through their roots. Which are a complex mixture of organic compounds secreted by plant roots, including sugars, amino acids, organic acids, fatty acids, vitamins, nucleotides, and various secondary metabolites [34]. PGPM can consume boundary cells including those composed of cellulose in plant roots through a combination of enzymatic degradation, invasion strategies, and symbiotic or pathogenic interactions [35,36]. They help in the decomposition of plant biomass, releasing nutrients that can be reused by plants and other organisms. PGPM responds to plants by accumulating nutrients through solubilization, phosphorus and potassium solubilizations, and nitrogen fixation and the production of plant hormones in the rhizosphere, which support plant health to promote plant growth [37,38] (Figure 1).

In this review, we focused on the role of rhizosphere microorganisms in enhancing plant health by promoting nutrient availability and bolstering plant immune responses against pathogen attacks. This overview summarizes the latest research on how host plants interact with rhizosphere microorganisms, highlighting their distinct regulatory mechanisms in response to stimulation, and was primarily conducted via searches on multiple platforms to gather relevant scientific publications.

## 2. Soil Macronutrients

Major macronutrients include phosphorus (P), potassium (K), and nitrogen (N), which are necessary for plants and animals in huge volume and their functions are non-replaceable by other elements [39,40]. Plants are dependent on the soil microbe community, which can possess the metabolic machinery to generate the nutrients in the soil by mineralizing and breaking down the organic forms of N, P, and K to obtain nutrients [41,42]. The microbial cells are unable to transport the long hydrophobic polymer chains directly into their cells through plasma membranes. They released extracellular enzymes bond to the polymer, initiating its breakdown via hydrolysis, which can occur through enzymatic modification, cell lysis, or as a result of the activities and nutrient cycling associated with protozoan predation [43,44]. These processes discharge inorganic N, P, and K forms to ionic species, as well as ammonium, nitrate, phosphate, and sulfate, which are available forms for plants to uptake [45].

Nitrogen (N) is essential for the synthesis of chlorophyll, the molecule primarily responsible for the green pigmentation in plants and central to the process of photosynthesis, which allows plants to convert light energy into chemical energy. Chlorophyll molecules are characterized by a complex structure known as a chlorin ring, at the heart of which are four nitrogen-containing pyrrole rings As a vital macronutrient, nitrogen significantly influences the growth and development of plants, being a key component in the processes related to energy metabolism, which are fundamental for maintaining plant vitality and growth. This element’s importance extends to its structural role in various biological molecules. For instance, nitrogen is a key component in amino acids, the building blocks of proteins. Proteins, in turn, are necessary for numerous plant functions, including enzyme activities, cellular structure, and the regulation of metabolic processes [46]. Additionally, nitrogen forms an integral part of nucleic acids, such as deoxyribonucleic acid (DNA) and ribonucleic acid (RNA), which are essential for genetic information storage and transmission in plants. It achieves this through its presence in nitrogenous bases, which are the fundamental units of these nucleic acids [47,48]. Plant can up taken the N only 30–50% of total N- fertilizers-compounds input to crops; the remainder is leached to groundwater or lost in the atmosphere [49]. Microbially-mediated processes regulate the balance between nitrogenous compound mineralization and immobilization as well as convert N fertilizers to ammonium (NH_4_^+^) and nitrate (NO_3_^−^), which are available forms for plant uptake such as *Bacillus velezensis*, *Pseudomonas stutzeri*, *Bradyrhizobium japonicum*, etc. [50,51]

The symbiotic relationship between legumes and rhizobia bacteria is one of the most studied models in nitrogen fixation, demonstrating a complex and mutually beneficial interaction between plants and microorganisms [52,53,54]. This interaction commences when rhizobia bacteria in the soil respond to specific chemical signals released by the roots of a legume plant [55]. These signals, primarily flavonoids, induce the bacteria to produce Nod factors (lipo-chitooligosaccharides), key signaling molecules that initiate the infection process [55]. The bacteria then infect the root hairs of the legume, causing the root hair to curl. This is followed by the formation of a new structure called a nodule within the root [56,57]. Inside this nodule, the bacteria multiply and convert atmospheric nitrogen (N_2_) into ammonia (NH_3_) through a series of enzymatic reactions involving the enzyme nitrogenase. This enzyme functions effectively in low-oxygen conditions, which the nodule provides. The study of Cui et al. [58] reported the induced organs on plant roots known as haustoria in plant parasitism and nodules in plant symbiosis are vital locations for nutrient uptake. Parasitic plants use an invasive organ known as a haustorium to exploit their host plants, while nodules, primarily forming in the roots of legumes, are a distinctive feature of their symbiotic relationship with nitrogen-fixing bacteria. In legume roots, nodules develop as lateral organs in reaction to nod factors. The detection of these nod factors by lysine motif (LysM) receptor kinases triggers a series of signaling events in the root epidermis. Following this, cortical cells undergo dedifferentiation and initiate cell division anew, leading to the formation of nodule primordia [58,59]. Inside the nodules, the bacteria are released and transformed into bacteroids, capable of fixing atmospheric nitrogen via the enzyme nitrogenase. The legume plant, in return, supplies the bacteria with carbohydrates and other organic compounds, which are essential for the energy needs of the bacteria [60]. The fixed nitrogen, now in the form of ammonia, is assimilated by the plant and utilized to synthesize proteins, nucleic acids, and other vital compounds. The ammonia produced by the bacteroid is then assimilated by the plant cells into organic compounds. The primary pathway involves the enzymes glutamine synthetase and glutamate synthase, which incorporate the ammonia into amino acids. These amino acids are then used for the synthesis of proteins and other nitrogen-containing compounds essential for plant growth and development (Table 1).

Phosphorus is required for the formation of sugar phosphates for respiration and energy transfer processes, adenosine triphosphate (ATP), and photosynthesis activity [61,62]. Phosphate solubilizing microorganisms (PSMs), including species of bacteria, fungi, and actinomyces, are a large microflora that mediate bioavailable soil P through mineralizing organic P [63]. A large portion of the phosphorus in the soil is present in insoluble forms that the plants are unable to uptake. PSMs alter the soil’s pH to help inorganic phosphates become soluble, which plants can uptake. In alkaline soils, PSMs secrete organic acids such as gluconate, citrate, lactate, and succinate, which lower pH and dissolve calcium phosphate (Ca_3_(PO_4_)_2_). Whereas in acid soils, PSMs generate protons and phosphohydrolases to mineralize and solubilize the various P forms in soils [64,65]. *Bacillus* sp., *Pseudomonas* sp., *Rhizobium* sp., and *Aspergillus* sp. can convert insoluble phosphorus into soluble forms such as monobasic (HPO_4_^2−^) or dibasic phosphate (H_2_PO_4_^−^), which function as biofertilizers. The primary method employed by PSMs is the production of organic acids [66]. As part of their metabolic activities, these microbes secrete organic acids, which acidify their immediate environment. The acidification process involves chelation, where organic acids bind to and sequester cations like calcium, iron, and aluminum that are attached to phosphate, effectively freeing the phosphate ions [67,68]. Additionally, these acids can compete with phosphate for adsorption sites in the soil, further facilitating the release of phosphate. Another crucial mechanism is the enzymatic breakdown of organic phosphorus compounds. This process, termed mineralization, is vital for making organic phosphorus available to plants. Acidolysis is also a significant process, particularly in the breakdown of mineral phosphates. This involves the direct action of hydrogen ions, released from the organic acids, on mineral phosphates, leading to their dissolution into more soluble forms. Protonation and ion exchange reactions further aid in this process. The increase in hydrogen ions in the soil from microbial activity can replace the cations bound to phosphate, enhancing phosphate solubilization [69]. In addition, some microbes produce siderophores, which are special compounds that chelate metals, including those bound to phosphates. This chelation can indirectly contribute to making phosphate more soluble. Pan et al. [70] describe the ability of PSMs, They have the capacity to release various low-molecular-weight organic acids like malic, succinic, fumaric, acetic acid, tartaric acid, malonic acid, glutamic acid, propionic acid, butyric acid, lactic, 2-ketogluconic, saccharinic, and oxalic acids as they grow. These specific organic acids possess the ability to form chelates under conditions of low pH, utilizing their hydroxyl and carboxyl groups to bind with metal ions found in the soil, including Fe^3+^, Al^3+^, and Ca^2+^ [71]. Notably, their chelation with Ca^2+^ is particularly significant, leading to competition with phosphates for adsorption sites in the soil. This competition ultimately enhances the soil’s ability to absorb phosphate, augmenting the solubilization capacity of inorganic phosphorus [68,72]. Consequently, this process increases the solubility and accessibility of mineral phosphates. The efficiency of solubilization is also influenced by the type of organic acids involved, with tricarboxylic and dicarboxylic acids generally proving more effective compared to mono- and aromatic acids. Additionally, aliphatic acids tend to exhibit greater effectiveness in solubilizing phosphates when compared to phenolic, citric, and fumaric acids (Table 1).

Potassium (K) is crucial for metabolism, enzyme activation, osmoregulation, charge balance, preventing excessive water loss, and regulating plant stomatal movement [73]. Abnormal growth and development caused by a K deficit have a direct impact on crop output and disease resistance. In soils, potassium solubilizing microbes (KSMs) help mobilize K from the soil or mineral to plants [74]. Different organic acids secreted by KSMs cause minerals to release K, making it accessible to plants [75]. Many bacteria, such as *Bacillus* spp., *Pseudomonas* sp., *Pantoea agglomerans*, and *Halomonas aquamarina*, have capacity to solubilize K minerals. The mechanism through which soil microorganisms assist in the solubilization and mobilization of potassium, a crucial nutrient for plant growth, highlights the intricate relationships within soil ecosystems [76]. Potassium in soil exists in various forms, with a significant portion locked in mineral structures that plants cannot directly use. Soil microorganisms, particularly certain bacteria and fungi, are adapt at transforming this unavailable potassium into accessible forms for plants [77]. These microorganisms utilize their metabolic capabilities to produce organic acids, which play a central role in potassium solubilization. The acids released by microbes acidify their surrounding soil environment. This acidification enhances the solubility of potassium minerals by lowering the pH. Additionally, the acids can chelate or form complexes with the cations associated with potassium in soil minerals, thereby releasing potassium ions into the soil solution [73]. Beyond solubilization, microbes also contribute to the mobilization of potassium through ion exchange processes. The microbial biomass acts as a cation exchange reservoir, releasing potassium ions into the soil solution [78]. The interaction between microorganisms and plant roots also plays a significant role in this process. Root exudates, consisting of various organic compounds, stimulate microbial activity in the rhizosphere (the soil region near the roots). K^+^-rich minerals undergo transformation into plant-accessible forms through three key mechanisms. Firstly, acidification plays a primary role as it breaks down the potassium aluminosilicate complex by releasing either organic or inorganic acids. These transformation results in potassium silicate or aluminum silicate becoming available to plants. Secondly, siderophores are produced, forming chelating compounds with Fe^+2^, Si^+4^, Ca^+2^, and Al^+3^, ultimately releasing K^+^ into the exchangeable K pool [79,80]. Thirdly, certain extracellular polymers, such as proteins or EPS, are synthesized by microorganisms [81]. These microbes create biofilms around mineral rocks and undergo weathering processes, altering the rocks’ morphology and, in the process, making K^+^ accessible to plants [82]. This enhanced microbial activity leads to increased potassium solubilization and mobilization. The soluble potassium is then absorbed by the plant roots, facilitated by specialized potassium transporters in the root cells. This enhanced availability of potassium due to microbial activity is crucial for plant health. Therefore, the role of soil microorganisms in making potassium available directly impacts plant growth and health, emphasizing the importance of these microorganisms in maintaining soil fertility and supporting robust plant development (Table 1).

Furthermore, the composition and function of microbial communities are significantly influenced by various environmental factors, including temperature, pH, moisture content, oxygen availability, and nutrient availability [83]. These factors not only determine the types of microorganisms that can thrive in a particular environment but also shape the interactions within microbial communities and between microbes and their environment. Temperature is a critical environmental factor that influences microbial metabolism, growth rates, and community structure [84]. The optimal temperature range for microbial activity significantly affects the decomposition rates of organic matter, the cycling of nutrients, and the overall productivity of ecosystems [85]. Water availability is essential for microbial life, as it facilitates the mobility of nutrients and enzymes required for microbial metabolism. In addition, soil environments with high moisture content support a diverse and active microbial community, crucial for processes such as decomposition, nutrient mineralization, and the formation of soil aggregates. The pH level of an environment, indicating its acidity or alkalinity, is another major determinant of microbial diversity. Different microorganisms have adapted to thrive at specific pH levels, ranging from highly acidic to highly alkaline conditions. The pH of a habitat can influence microbial processes such as nitrification and denitrification, which are essential for nitrogen cycling in ecosystems [86,87].

**Table 1:** Effects of plant growth promoting microorganisms (PGPM) on crops production.

Microorganisms	Plant	Mechanisms	Beneficial Effects	References
*Flaisolibacter gensengsoil* Williams 82	Soybean	Nitrogen fixation, reduced the stress of molybdenum nanoparticles	Improved growth; roots, stems, leaves, nodules	[88]
*Bacillus velezensis* S141	Soybean	Nitrogen fixation, auxin, cytokinin biosynthesis	Improved growth; nodule numbers, nodule dry weight, root-shoot dry weight, plant dry weigh	[89]
*Bacillus pumilus*	Tomato	Nitrogen fixation, Improved N uptake	Improved growth; shoot fresh weight, shoot dry weight, root fresh weight, root dry weight	[90]
*Pseudomonas stutzeri* A1501	Maize	Nitrogen fixation, ammonia oxidizers	Improved growth; shoot, root, yields	[91]
*Pseudomonas stutzeri Spirulina platensis extract*	Onion	Nitrogen fixation, Hydrogen cyanide, indole acetic acid, ammonia, pectinase activity	Improved growth; marketable yield, total bulb yield, bulb weight, bulb diameter	[92]
*Pseudomonas aeruginosa* CQ-40	Tomato	Nitrogen fixation, Phosphates solubilization, cellulase, protease, ferrophilin, antibacterial metabolites,	Promote Growth; plant height, stem thickness, dry and fresh weight, main root length and Control *Botrytis cinerea*	[93]
*Bradyrhizobium* sp. A2-10, *Pseudomonas* sp. A6-05	Calopo	Nitrogen fixation, Phosphates-solubilization, siderophore production, indole acetic acid	Dry mass of shoots (SDM) and roots (RDM) Nodules	[94]
*Lysinibacillus varians* KUBM17	Radish	Nitrogen fixation, Phosphates solubilization, siderophore production, indole acetic acid	Plant growth under cadmium stress condition.	[95]
*Bradyrhizobium japonicum* SAY3-7, *Bradyrhizobium elkanii* BLY3-8, *Streptomyces griseoflavus*	Mung Bean, Cowpea, Soybean	Nitrogen fixation, nitrogen, phosphorus, and potassium uptake	Plant growth, nodulation, yield components	[96]
*Arthrobacter* sp. V54, *Bacillus* sp. V62	Tomato	Phosphates solubilization	Promote Growth; plant height, shoot, and root dry weights,	[97]
*Aspergillus niger*, *Purpureocillium lilacinum*, *Bacillusamyloliquefaciens*, *Paenibacillus polymyxa*	Apple	Increased uptake N, P, K, Mg, and Ca	Increase of N, P, K, Mg content in apple orchard soil	[98]
*Klebsiella variicola*	Jerusalem artichoke	Phosphates solubilization	Improved growth/Tuber	[99]
*Pseudomonas* sp.	Wheat	Phosphates solubilization	Improved root and shoot dry matter, grain yield,	[100]
*Staphylococcus* sp.	Banana	Phosphates solubilization	Promote plants growth	[101]
*Citrobacter amalonaticus*, *Bacillus safensis*	Maize	Phosphates solubilization	Promote plants growth	[102]
*Bacillus licheniformis*	Rice	Phosphates solubilization	Promote plants growth	[103]
*Paenibacillus mucilaginosus*	Apple	Potassium solubilization	Promote Growth; seedlings	[104]
*Pantoea agglomerans*, *Rahnella aquatilis*, *Pseudomonas orientalis*	Rice	potassium solubilization	Improved grain yield	[105]
*Bacillus* sp. MN54	Pearl millet	enhance dry matter remobilization and translocation to the grains	Improved grain yield; Grains Per Ear, grain weight, Improved protein, iron and zinc concentration in pearl millet seeds	[106]
*Bacillus megaterium*, *Bacillus mucilaginous*	Chili pepper	Potassium solubilization, soil organic carbon, total nitrogen, available Phosphorus	Promote Growth; Fruits, Shoots, Roots	[107]
*Halomonas aquamarina* EU-B2RNL2, *Pseudomonas extremorientalis*	Chili	Potassium solubilization, phosphates solubilization	Shoot/root biomass and length, number of leaves, branches and fruits per plant	[108]

## 3. Induced Plant Defense

PGPMs also act directly-indirectly natural pesticide as well as biocontrol agents inhibiting plant pathogens through hyper parasitism, competition for nutrients, antagonism by production of antibiotics substances, and induces systemic resistance [109]. The diverse microbial ecosystem of the plant rhizosphere increases the disease resistance of plants via activation of dormant plant defense systems or defensive priming [33]. In addition to activating defensive priming, they regulate defense signaling pathways that result in the creation of secondary metabolites. In PGPMs-colonized plants, the activation of induced systemic resistance frequently confers a greater capacity for self-defense (ISR), however in several study reported it can also activation by systemic acquired resistance (SAR).

Priming is a mechanism that leads to a physiological state that enables plants to rapidly respond to biotic or abiotic stress. This is an adaptive strategy due to being inactivated, or slightly activated during a given priming stimulus as well as a mild level of biotic or abiotic stress, whereas a stronger response is activated during pathogen or insect invasion (secondary stimulus) [110].

The mechanism of priming includes all the events from perception of stimuli by the plant to all physiological, molecular, and epigenetic changes which take place in the plant, leading to enhanced readiness of the plant to combat future challenges.

ISR refers to types of induced resistance in which plant defenses are conditioned by a previous infection [111]. Its mechanism of action does not rely on direct killing or suppression of the invading pathogen, but rather on boosting the immune system’s resistance by increasing molecular, phytohormone, chemical barrier, epigenetic changes or physical properties of the host plant that specifically target the pathogens [112]. ISR can be enabled by the rhizosphere PGPMs through a root signal influencing different inducers, as well as lipopolysaccharides (LPS), volatile organic compounds (VOCs), flavonoids, and hormones. These can affect or inhibit pathogens, insect herbivores and insect natural enemies. A plant’s system triggers an innate response in the state in which it experiences higher resistance to host pathogen activity. Expression of ISR, the mechanism by which pathogens such as viruses, bacteria, or fungi initiate infection, can induce a series of localized reactions in and around infected host cells. Plants cells response to the pathogens by produce reactive oxygen species (ROS), superoxide anion (O^−2^) and hydrogen peroxide (H_2_O_2_). These event call hypersensitive responses (HR). HR is a form of the rapid death cell (within 24 h) at the spot of pathogen invasion. The pathogen is restricted to the dead cells and cannot spread beyond the site of initial infection. The activation of the HR results in the production of antimicrobial substances, such as hormones, phytoalexins, and pathogenesis-related (PR) proteins. Phytoalexins are mostly associated with the local reaction, whereas PR proteins can be found both locally and systemically [113]. PR proteins are generally used as a key marker of plant immune system [114]. It is well-established that PGPMs can transmit resistance to pests and diseases through ISR. Plant growth-promoting rhizobacteria (PGPR) and plant growth-promoting fungi (PGPF) have been demonstrated to increase plant defensive capacity as well as their ability to stimulate SA-dependent ISR responses, which are familiar to the SAR [115]. Applications of PGPMs can activate plant defense via the SAR pathway in a variety of plants by boosting salicylic acid (SA) and PR-protein levels, which have detrimental effects on pathogens for example, *Bacillus subtilis* stain BU412 induced tobacco plants show higher levels of defense compounds via increased superoxide dismutase (SOD), peroxidase (POD), polyphenol oxidase (PPO), and phenylalanine ammonia lyase (PAL) resulted in tomato developing resistance to *Pseudomonas syringae* attack [116]. *Bacillus vallismortis* stain TU-Oraga21 produced surfactin and iturin A to inhibit spore germination of *Magnaporthe oryzae*, the causal agent of rice blast at the infection court and accumulated endogenous salicylic acid, phenolic compounds, and hydrogen peroxide (H_2_O_2_) during the infection process to eliminates the pathogen [117]. *Pseudomonas fluorescens* SP007s enhanced immune of kale and soybean by accumulation of endogenous salicylic acid and phenolic compounds against *Pectobacterium carotovorum*, the causal agent of soft rot in kale [118] and *Xanthomonas axonopodis* pv. *glycines*, the causal agent of pustule disease in soybean [119]. Also, *P. fluorescens* SP007s plays role of carAB that encoded carbamoylphosphate synthetase (CPSase) to degrade a diffusible signal factor (DSF) of *X. axonopodis* pv. *glycines* [119].

To date, diverse PGPM strains have improved the defensive capacity of plants through the combination of ISR and SAR against pathogens that are resisted through both pathways, besides extending protection to a broader spectrum of pathogens than ISR or SAR alone (Table 2). The combined activation of ISR and SAR in plants by PGPMs represents a sophisticated enhancement of the plant’s defensive capabilities. When PGPMs strains induce both of these pathways, they create a synergistic effect that offers broad-spectrum defense against a range of pathogens. In this combined state, ISR acts as a rapid response mechanism [120]. It primes the plant’s defense systems, preparing them to react swiftly and effectively upon pathogen attack. This priming means that while the full defense responses are not immediately activated, they are on standby to respond more robustly when needed. This aspect of ISR is particularly crucial for dealing with initial pathogen attacks and providing localized protection. On the other hand, SAR provides a more sustained and systemic form of protection [121]. Triggered by PGPMs in a manner similar to how it responds to a pathogenic attack, SAR involves the accumulation of salicylic acid, which activates a wide array of defense genes throughout the plant. This response is not limited to the site of PGPMs interaction but extends to the entire plant, offering long-lasting protection against subsequent pathogenic invasions. The interaction between and SAR in plant defense involves cross-linking and cross-talk [122,123]. When a plant is exposed to ISR-inducing rhizobacteria, it can activate ISR, resulting in the production of defense-related compounds [124]. Later, if the same plant encounters a foliar pathogen, it can also activate SAR, which produces chemicals like SA for resistance [125]. The cross-linking occurs when ISR-inducing rhizobacteria stimulate the production of SA or other signaling molecules associated with SAR, thereby enhancing the plant’s SAR response against the foliar pathogen. Cross-talk between ISR and SAR involves the communication or exchange of signals between different defense pathways or components within the plant [126]. For example, ISR induction by rhizobacteria may lead to the production of jasmonic acid (JA), which is typically associated with defense against herbivores and necrotrophic pathogens. SAR primarily uses SA as a signaling molecule. Cross-talk can happen when JA from ISR interacts with the SA pathway of SAR [127,128]. This interaction allows the plant to balance the activation of both ISR and SAR based on the specific threat it faces.

A specific role of the metabolites produced by several groups of PGPR in ISR and SAR is their ability to act as signaling molecules that trigger the plant’s defense mechanisms. These metabolites can include compounds such as, VOCs, siderophores, antibiotics, phytohormones, and exopolysaccharides [129,130]. For example, dimethyl disulfide (DMDS) is a microbial VOC produced by bacteria such as *Bacillus* species. DMDS contributes to the characteristic smell of soil and is involved in signaling and defense mechanisms in several plants such as potato, rice, tomato, and maize [131,132,133,134]. The study of Ayaz et al. [135] reported that nematicidal volatiles from *Bacillus atrophaeus* strain GBSC56 promote growth and stimulate induced systemic resistance in tomato against *Meloidogyne incognita* by increasing levels of SOD, CAT, POD, APX, PR1, PR5, and SlLOX1 to efficiently manage root-knot disease. Likely, several studies have shown that functions of VOC not only direct effect to pathogens but extend to induce resistance against plant pathogens and to promote plant growth.

**Table 2:** Effects of plant growth promoting microorganisms (PGPM) in induced systemic resistance.

Microorganisms/Agents	Plant	Target Pathogens	Indirect Mechanisms	References
*Aspergillus fumigatus Rhizopus oryzae*	Tomato	*Fusarium oxysporum*	Increased POD, PPO	[108]
*Trichoderma harzianum*, *Trichoderma asperellum Paenibacillus dendritiformis*	Chilli Pepper	*Colletotrichum truncatum*	Increased SOD, POX, PPO, CAT, APX, GPX, PAL	[136]
*Trichoderma hamatum* Strain Th23	Tomato	Tobacco Mosaic Virus	Increase PPO, CAT, SOD PR-1 and PR-7	[137]
*Bacillus amyloliquefaciens TBorg1*	Tomato	Tobacco Mosaic Virus	PPO, POX, C4H, HCT, and CHI PR-1 and PR-5	[138]
*Trichoderma harzianum*	Cucumber	*Fusarium oxysporum*	Regulating ROS and RNS Metabolism	[139]
*Trichoderma* strains viz., T-42, MV-41, DFL, and RO,	Chickpea	*Fusarium oxysporum f.* sp. *ciceris*	Lignifications	[140]
*Bacillus subtilis MBI600*	Tomato	*Rhizoctonia solani*, *Pythium ultimum*, *and Fusarium oxysporum f.* sp.	SiPin6 and SiLax4, PR-1A, GLUA, CHI3, LOXD, PAL	[141]
*Bacillus amyloliquefaciens* FZB42	Tomato	*Sclerotinia sclerotiorum*	GST, SOD, PAL, HMGR, and MPK3	[142]
*Bacillus amyloliquefaciens Aspergillus spinulosporus*	Rice	*Xanthomonas oryzae* pv. *oryzae*	SOD, PAL, PO, PPO TPC	[143]
*Bacillus vallismortis* TU-Oraga21	Rice	*Magnaporthe oryzae*	Increased salicylic acid, phenolic compounds, and hydrogen peroxide (H_2_O_2_)	[117]
*Pseudomonas putida* BP25	Rice	*Magnaporthe oryzae*	OsPR1.1, peroxidase and total phenol compound	[144]
*Pseudomonas fluorescens* SP007s	Kale Soybean	*Pectobacterium carotovorum* *Xanthomonas axonopodis* pv. *glycines*	Increased salicylic acid and phenolic compounds	[118,119]
*Trichoderma spp.*	Grapevine	*Plasmopara viticola*	PR-2, OSM2, HSR hypersensitive response	[145]
*Trichoderma asperellum*	Onion	*Sclerotium cepivorum*	AcPR1, AcPAL1	[146]
*Trichoderma* spp.	Soybean	*Fusarium virguliforme*	PR-3, PR-2, PR-12, CHS	[147]
*Trichoderma asperelloides*	Muskmelon	*Stagonosporopsis cucurbitacearum*	POD, PPO, chitinase, β-1,3-glucanase	[148]
*Azospirillum brasilense* REC3	Strawberry	*Macrophomina phaseolina*	Accumulation of ROS, salicylic acid, callose, lignin, the increase of biofilm formation on leaves, and stomatal closure	[149]

Footnote: POD: Peroxidase, PPO: Polyphenol Oxidase, SOD: Superoxide Dismutase, POX: Peroxidase), CAT: Catalase, APX: Ascorbate Peroxidase, GPX: Glutathione Peroxidase, ROS: Reactive Oxygen Species, PAL: Phenylalanine Ammonia-Lyase, PR-1A: Pathogenesis-Related Protein 1A, PR-1: Pathogenesis-Related Protein 1, PR-2: Pathogenesis-Related Protein 2 (often Beta-1,3-Glucanase), PR-3: Pathogenesis-Related Protein 3 (often Chitinase), PR-5: Pathogenesis-Related Protein 5 (often Thaumatin-like proteins), PR-7: Pathogenesis-Related Protein 7 (often Endoproteinase), PR-12: Pathogenesis-Related Protein 12, C4H: Cinnamate 4-Hydroxylase, HCT: Hydroxycinnamoyl-CoA Shikimate/Quinate Hydroxycinnamoyltransferase, CHI: Chitinase, RNS: Reactive Nitrogen Species, SiPin6: proteins involved in auxin transport, SiLax4: gene or protein related to auxin influx, GLUA: Glutamate receptor, CHI3: Chitinase 3, LOXD: Lipoxygenase D, GST: Glutathione S-Transferase, HMGR: 3-Hydroxy-3-Methylglutaryl-CoA Reductase, MPK3: Mitogen-Activated Protein Kinase 3, OSM2: Osmotic Stress, HSR: Heat Shock Response, AcPR1: pathogenesis-related protein 1, AcPAL1: Phenylalanine ammonia-lyase 1 from “), CHS: Chalcone Synthase.

## 4. Rhizosphere Communication and Signaling for Soil Health

Soil health is essential for sustainable agriculture, ecosystem functioning, and environmental stewardship. Monitoring and improving soil health contribute to long-term productivity, environmental sustainability, and the ability of the soil to provide essential ecosystem services that sustain plants, animals, and humans and connect agricultural [150]. Sustainable soil management practices aim to enhance and maintain soil health, recognizing the interconnectedness of physical, chemical, and biological aspects of soil. Rhizosphere microorganisms play a crucial role in improving soil health through various mechanisms that enhance nutrient availability, organic matter decomposition, disease suppression, and overall ecosystem functioning. Communication and signaling among rhizosphere microorganisms and plants play a crucial role in soil health. These interactions involve the exchange of chemical signals and responses that influence the activities of both microorganisms and plants. The communication and signaling mechanisms in the rhizosphere contribute to nutrient cycling, plant growth promotion, disease resistance, and overall soil health [151]. Root exudates play a crucial role in signaling and communication in the rhizosphere which contain a diverse array of organic compounds, including sugars, amino acids, organic acids, vitamins, and secondary metabolites. They serve as chemical signals that attract and stimulate specific microorganisms in the rhizosphere. For example, plants release specific compounds to attract mycorrhizal fungi, which form symbiotic relationships with plant roots and enhance nutrient uptake [13]. Microorganisms in the rhizosphere have evolved to recognize and respond to the chemical composition of root exudates. They can sense these signals and adjust their metabolic activities and behavior accordingly [152,153]. For example, some rhizosphere microorganisms modulate their metabolic activities through quorum sensing (QS), a mechanism by which they communicate with each other using signaling molecules [128]. This sophisticated communication system allows rhizosphere microorganisms to sense changes in their population density and coordinate their metabolic activities accordingly, involves the production and release of signaling molecules called autoinducers. As microorganisms grow and multiply, they continuously release these autoinducers into their environment. When the concentration of autoinducers reaches a critical threshold, it triggers a coordinated response within the microbial community. They can modulate their metabolic activities through QS when facing plant defense challenges [154]. This includes adjusting secondary metabolite production, nutrient acquisition strategies, biofilm formation, and expression of virulence factors [155]. As the population density of microorganism’s increases, QS triggers changes in gene expression and metabolic activities. Upon detecting a sufficient concentration of signaling molecules, bacteria activate specific gene expression pathways. These pathways regulate the production of various extracellular products, enzymes, or other factors that contribute to cooperative behaviors. This coordination allows microorganisms to behave collectively in response to environmental conditions such as biofilm formation, soil structure, bioremediation, and ecosystem resilience [156,157,158]. N-acyl homoserine lactones (AHLs) are indeed a well-documented class of QS molecules. Several studies suggested that AHLs can elicit changes in defense mechanisms. Shrestha et al. [159] reported the pretreatment with AHL is associated with increased barley MAP kinase activation and defense-related PR1 and PR17b gene regulation. Activation of MAP kinases and PR-gene are a common response in plants during defense signaling pathways.

Microbial–plant interactions represent a crucial aspect of plant biology, encompassing a wide range of relationships that can be beneficial to the plant. These interactions are mediated by various metabolites produced by the microbes, which can significantly influence plant growth, health, and response to environmental stresses [160]. One of the most well-known examples of beneficial microbial–plant interactions is the symbiotic relationship between nitrogen-fixing bacteria and leguminous plants. Rhizobia, a group of soil bacteria, can establish a symbiotic relationship with the roots of legume plants, such as peas, beans, and clovers [52]. The process begins when flavonoids secreted by the plant roots attract the rhizobia. In response, the bacteria produce nod factors (lipo-chitooligosaccharide signaling molecules) that trigger root hair deformation and the formation of an infection thread in the host plant [161]. This interaction leads to the formation of root nodules, specialized plant organs where the bacteria convert atmospheric nitrogen into ammonia, a form of nitrogen that plants can assimilate and utilize for growth. This nitrogen-fixing symbiosis is critical for the global nitrogen cycle and enables legumes to grow in nitrogen-poor soils, reducing the need for synthetic nitrogen fertilizers. Besides nitrogen fixation, microbial metabolites play a key role in signaling and plant growth promotion. For instance, certain strains of PGPR can produce phytohormones such as auxins, cytokinins, and gibberellins [162]. These hormones can directly influence plant growth by enhancing root development, which increases the plant’s ability to uptake water and nutrients from the soil. An example of this is the bacterium *Azospirillum brasilense*, which can produce indole-3-acetic acid (IAA), a type of auxin [163]. When *A. brasilense* colonizes the roots of grasses and cereals, the IAA it produces can stimulate root elongation and branching, leading to increased root surface area and enhanced mineral and water uptake by the plant. Furthermore, Microbial metabolites can also trigger the plant’s innate immune system. For example, certain strains of *Pseudomonas fluorescens* and *Bacillus amyloliquefaciens* produce metabolites that can induce ISR and SAR in plants [126]. When applied to roots, these beneficial microbes can prime the plant’s systemic defense mechanisms, enabling it to more effectively fend off a wide range of pathogens.

**Figure 2:**
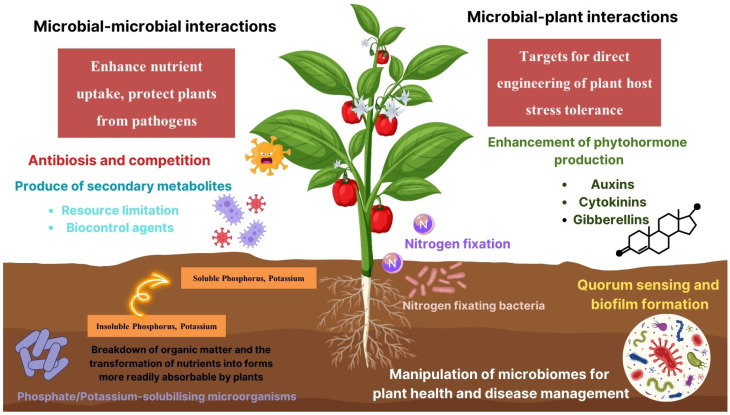
Schematic overview of microbial–plant and microbial–microbial interactions in plant health.

Microbial–microbial interactions are a fundamental component of microbial ecology, influencing community structure, function, and dynamics [164]. These interactions can range from mutualistic and symbiotic to competitive and antagonistic, with microbes producing a variety of metabolites that mediate these relationships [165]. One of the most studied aspects of microbial–microbial interactions is antibiosis, where one microorganism inhibits or kills another through the production of antimicrobial compounds. A classic example of this is the production of antibiotics by soil bacteria of the genus *Streptomyces*. Streptomyces are renowned for their ability to produce a wide array of secondary metabolites, including antibiotics like streptomycin and tetracycline [166]. These compounds play a significant role in the soil microbial ecosystem by mediating competitive interactions. Streptomyces species use these antibiotics to suppress competing microorganisms in the soil, thereby securing resources and space for themselves [167]. Quorum sensing is another pivotal mechanism in microbial–microbial interactions, involving the production and detection of signaling molecules known as autoinducers [168]. This process allows microbial populations to coordinate their behavior in response to population density. Syntrophy, or cross-feeding, is a mutualistic interaction where one microorganism consumes a product generated by another, often leading to enhanced survival and growth for both parties [169]. An example of syntrophy is observed in the breakdown of complex organic compounds in anaerobic environments, such as in methanogenic ecosystems. In these systems, certain bacterial species degrade complex polymers (like cellulose) into simpler compounds such as organic acids and alcohols, which they cannot completely metabolize under anaerobic conditions. Methanogenic archaea then consume these intermediates, producing methane as a byproduct [170]. This interaction is critical for the stability and function of anaerobic digesters and natural methanogenic environments. Biofilms are complex communities of microorganisms attached to surfaces, encapsulated within a self-produced matrix of extracellular polymeric substances. Microbial–microbial interactions within biofilms are highly dynamic, involving competition for nutrients and space, as well as cooperation for biofilm formation and defense against external threats. For example, in mixed-species biofilms, some bacteria produce extracellular enzymes or metabolites that can be utilized by other members of the community, enhancing the overall resilience and metabolic efficiency of the biofilm [171]. Through these mechanisms, microbial–microbial interactions play a critical role in shaping the health, growth, and defense of plants (Figure 2).

## 5. Advances in Microbiome Research and Omics Technologies

Microbiome research has become a focal point in understanding the intricate interactions between plant root systems and their surrounding soil microorganisms. The rhizosphere is a dynamic soil zone influenced by plant roots, teeming with a diverse array of microorganisms, including bacteria, fungi, and archaea [172]. This microbial community is shaped by various factors like plant species, soil type, and environmental conditions, leading to unique interactions for each plant species. A significant aspect of rhizosphere microbiome research focuses on how these microbes contribute to nutrient cycling, particularly for essential nutrients like nitrogen and phosphorus. Some microbes have the ability to fix atmospheric nitrogen or solubilize phosphorus, making them more accessible to plants. Additionally, these microorganisms can defend plants against pathogens by producing antimicrobial substances or triggering systemic plant resistance mechanisms. Thus, microbial communities and microbes have beneficial effects on their host plant. Multiple studies have documented the advantages of microbial communities in promoting plant growth and development. These microorganisms aid in developing tolerance against both biotic and abiotic stressors. The plant microbiome exerts a significant influence on both the health and productivity of plants [173]. The impact of the microbiome on plant health, particularly in the context of *Arabidopsis thaliana*, a model organism for plant biology, has been extensively studied and reveals complex interactions [174,175,176,177]. These studies have shown that healthy plants host diverse and structured communities of microorganisms that colonize almost every part of the plant [177,178,179]. This plant-associated microbiome offers significant advantages to the host, including promoting growth, aiding in nutrient uptake, enhancing stress tolerance, and providing resistance to pathogens [180]. Omics technologies, such as metagenomics, metatranscriptomics, and metaproteomics, are playing a pivotal role in advancing our understanding of microbial communities and their interactions within various ecosystems [181]. These technologies facilitate the study of microbial interactions, such as cross-feeding, competition, and cooperation, by analyzing gene expression patterns and metabolic networks within microbial consortia. They have revolutionized our understanding of microbiomes across diverse ecosystems. Understanding these interactions is crucial for deciphering the ecological and physiological dynamics of microbial communities. In a study of the rhizosphere microbiome of a crop plant, metagenomics can reveal the presence of specific bacterial species, such as nitrogen-fixing rhizobium, and their genes responsible for nitrogen fixation, providing insights into how the microbiome contributes to plant nutrient acquisition [182]. A research study reported by Tian et al. [183] investigated the root-associated microbiome of healthy and nematode-infected tomato plants. They provide that the bacterial populations enriched in the root gall exhibited a high abundance of genes associated with the breakdown of plant polysaccharides, as well as with carbohydrate and protein metabolism, along with genes involved in biological nitrogen fixation using function-based metagenomic analysis. Furthermore, metagenomic analysis and the comparative study of plant-associated microbiomes have yielded innovative insights into the phylogenetic diversity and functional dynamics of the plant microbiome, as well as their intricate interactions with host plants [184]. Certain microbial groups, drawn from soil reservoirs into the rhizosphere, seem to establish a unique core microbiome. This core set of microbes around the roots plays a vital role in enhancing plant growth and overall health. So far, the identification of the core microbiome associated with plants, whether located in the rhizosphere, endosphere, or phyllosphere, has mainly relied on taxonomic indicators. The network analyses provide a comprehensive view of the taxonomic and functional landscape of microbial communities, surpassing the insights gained from traditional, isolated studies of microbial taxa. Linking taxonomic data with functional gene information illuminated the roles of specific microbial taxa in processes such as nitrogen fixation, carbon sequestration, and pollutant degradation. The networks highlighted potential metabolic pathways facilitated by microbial interactions, underscoring the collaborative nature of microbial communities in ecosystem processes. Several factors may explain the dramatic variation in the correlation between microbial diversity and plant diversity. The plant-microbial relationship can vary among different microbial taxonomic groups. example, based on a site-level analysis, the richness of certain bacterial taxa, such as nitrogen-fixing bacteria, was more closely related to plant diversity, variety of plant species, than were other taxonomic groups [185].

This suggests that the diversity of plants or the unique characteristics of each plant species in an area may play a significant role in shaping the composition of microbial communities, particularly those bacteria that contribute to key ecological processes like nitrogen cycling. The relationship between plant diversity and the prevalence of nitrogen-fixing bacteria highlights the intricate interactions within ecosystems, where plant types can influence the microbial population dynamics and, in turn, the overall nutrient availability and soil health.

Kober et al. [186] found 206,596 prokaryotic and 53,710 fungal Operational Taxonomic Units (OTUs) in Styrian agricultural soils using 16S rRNA gene and fungal ITS region sequencing. Vineyard soils had higher fungal diversity and distinct fungal compositions than orchards and other agricultural fields, while prokaryote diversity was consistent. Soil pH strongly influenced microbial community structures in vineyards and orchards, demonstrating its edaphic modulation activity. Similarly, Wu et al. [187] reported that bacterial diversity in arable soils is strongly related to soil pH, with lower diversity under acidic and higher diversity under neutral pH conditions. The study also found that soil acid–base balance, sorption complex integrity, organic matter quantity and quality, and specific nutrient profiles affect microbial community topologies in agricultural environments. Prokaryotic and fungal communities responded differently to identical abiotic variables in orchard and vineyard environments. Vineyard microbiomes were more herbicide-resistant than orchard soils, which changed significantly. The abundance of Chthoniobacterales, Flavobacterium, and Monographella in orchard soils was significantly altered by herbicides. Nectria and Thelonectria, potentially pathogenic fungus, increased significantly in herbicide-treated orchard soils. These insights help refine soil management practices to conserve and improve agricultural soil microbiomes. Likely, the study of Dlamini et al. [188] demonstrated that the use of the metagenomic technique to examine the microbiome diversity of healthy and leaf blight infected maize rhizosphere. The study found that the maize rhizosphere microbiome’s ability to modify microbial functions to manage and sustain plant health depends on microbial diversity. The SEED subsystem database showed 12 bacteria, 4 archaea, and 2 fungus taxa prevalent across fields. Proteobacteria, Dienococcus-Thermus, Gemmatimonadetes, Chlorobi, Cyanobacteria, Planctomycetes, Verrucomicrobia, Acidobacteria, Firmicutes, Chloroflexi, and Bacteroidetes predominated. Archaea included Euryarchaeota, Thaumarchaeota, Crenarchaeota, and Korachaeota, although Ascomycota and Basidiomycota dominated. At phylum and genus level, beta analysis index showed significant difference in the abundance of the microbial community in healthy and leaf blight infected maize rhizospheres.

## 6. Conclusions and Future Perspectives

Rhizosphere microorganisms have the potential to offer more environmentally friendly ways to increase crop production. The study of the structural and functional traits of the beneficial microbes in diverse farm systems represents a discipline that is constantly developing on a global scale. All types of ecosystems benefit from having healthy soil microbial communities since they support a wide range of soil processes, including respiration, nutrient cycling, and soil properties. These benefits are achieved by managing their configuration, utility characteristics, biochemical-substrate, or enzymatic activity. Additionally, several stressors above and below ground impacted by the warmer environment have effects on soil organic matter, root exudation, and microbial community structure resulting in an increased incidence of pests and pathogens. In many cases, plant microbiomes (community of microorganisms) exert a positive function in the community by defending against pathogens, promoting growth and health, and providing plants with an advantage in terms of adaptation. Consequently, complex and dynamic interactions exist between plants and their microbiome, describing a plant–soil feedback mechanism that may be used to describe this connection and dependency between abiotic and biotic elements of the soil circumstance.

However, microbiome research still has a number of system-level knowledge gaps. Deciphering the intricate and dynamic microbial crosstalk in the soil system and the multitrophic interactions will undoubtedly be useful to farming systems, providing a fresh impetus to realize the full potential of the rhizosphere microorganisms. Therefore, in-depth understanding of their functions in the microbial–plant relationship, their community, their processes, or any requirements are needed. This knowledge will provide sustainable solutions by increasing soil fertility, disease tolerance, plant yield, and nutrition, as well as the potential for restoring degraded lands. This vast microbial world significantly contributes to the conservation of plant and human life on planet Earth to offer answers that aid in the creation of innovative biocontrol technologies and their applications.

## Data Availability

Not applicable.
